# (Cyanido-κ*C*)(2,2-di­phenyl­acetamido-κ^2^ *N*,*O*)bis­(η^5^-penta­methyl­cyclo­penta­dien­yl)zirconium(IV)

**DOI:** 10.1107/S1600536814001160

**Published:** 2014-01-22

**Authors:** Lisanne Becker, Anke Spannenberg, Perdita Arndt, Uwe Rosenthal

**Affiliations:** a Leibniz-Institut für Katalyse e. V. an der Universität Rostock, Albert-Einstein-Strasse 29A, 18059 Rostock, Germany

## Abstract

In the title compound, [Zr(C_10_H_15_)_2_(C_14_H_12_NO)(CN)], the Zr^IV^ atom is coordinated by two penta­methyl­cyclo­penta­dienyl ligands, the amidate ligand *via* the N and O atoms, and an additional C N ligand. The four-membered metallacycle is nearly planar (r.m.s. deviation = 0.008 Å). In the crystal, the mol­ecules are connected into centrosymmetric dimers *via* pairs of N—H⋯N hydrogen bonds.

## Related literature   

For structures of mononuclear group 4 metallocene complexes with *κ* ^2^-*N*,*O* chelating amidate ligands without additional coordination of its substituents, see: Arndt *et al.* (1996 ▶); Gambarotta *et al.* (1985 ▶); Haehnel *et al.* (2013 ▶); Ruck & Bergman (2004 ▶). For structures of group 4 metallocene complexes with *κ* ^2^ *N*,*O*-chelating OC(P)N(*R*) ligands, see: Segerer *et al.* (2000 ▶); Frömel *et al.* (2013 ▶). For a similar complex, see: Becker *et al.* (2013 ▶).
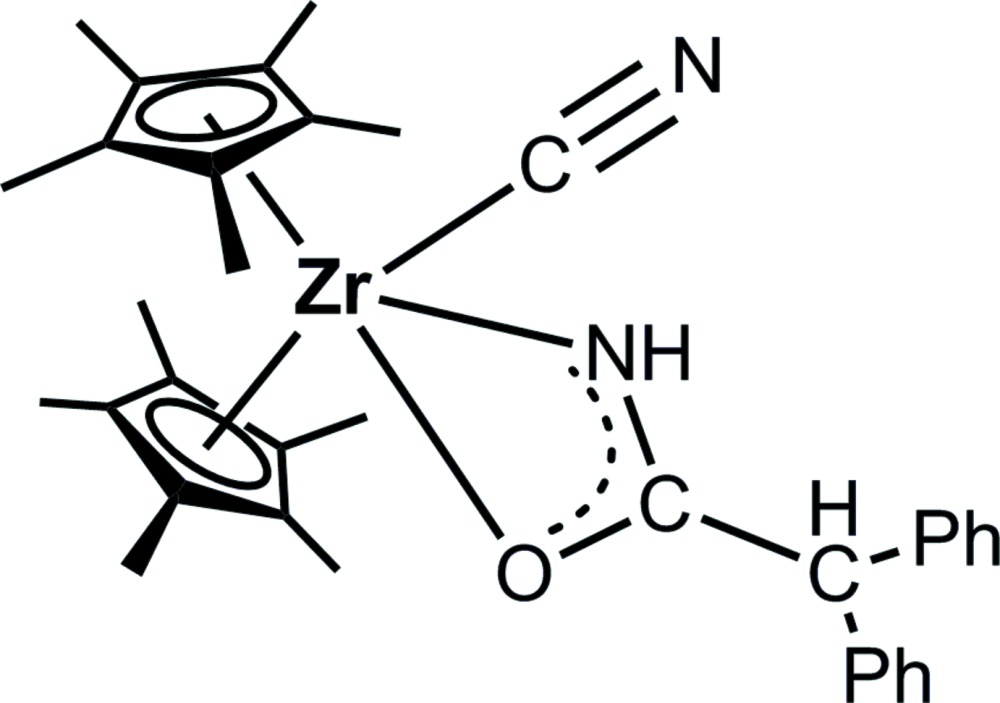


## Experimental   

### 

#### Crystal data  

[Zr(C_10_H_15_)_2_(C_14_H_12_NO)(CN)]
*M* *_r_* = 597.93
Monoclinic, 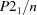

*a* = 11.8961 (3) Å
*b* = 12.0640 (3) Å
*c* = 21.2489 (5) Å
β = 97.036 (1)°
*V* = 3026.56 (13) Å^3^
*Z* = 4
Mo *K*α radiation
μ = 0.39 mm^−1^
*T* = 150 K
0.26 × 0.13 × 0.10 mm

#### Data collection  

Bruker Kappa APEXII DUO diffractometer
Absorption correction: multi-scan (*SADABS*; Bruker, 2008 ▶) *T*_min_ = 0.92, *T*_max_ = 1.00
82697 measured reflections
6612 independent reflections
5575 reflections with *I* > 2σ(*I*)
*R*_int_ = 0.047

#### Refinement  

*R*[*F* ^2^ > 2σ(*F* ^2^)] = 0.033
*wR*(*F* ^2^) = 0.085
*S* = 1.03
6612 reflections
370 parameters
1 restraint
H atoms treated by a mixture of independent and constrained refinement
Δρ_max_ = 1.11 e Å^−3^
Δρ_min_ = −0.46 e Å^−3^

### 

Data collection: *APEX2* (Bruker, 2011 ▶); cell refinement: *SAINT* (Bruker, 2009 ▶); data reduction: *SAINT*; program(s) used to solve structure: *SHELXS97* (Sheldrick, 2008 ▶); program(s) used to refine structure: *SHELXL97* (Sheldrick, 2008 ▶); molecular graphics: *SHELXTL* (Sheldrick, 2008 ▶); software used to prepare material for publication: *SHELXL97*.

## Supplementary Material

Crystal structure: contains datablock(s) I, global. DOI: 10.1107/S1600536814001160/bt6957sup1.cif

Structure factors: contains datablock(s) I. DOI: 10.1107/S1600536814001160/bt6957Isup2.hkl

CCDC reference:

Additional supporting information:  crystallographic information; 3D view; checkCIF report

## Figures and Tables

**Table 1:** Hydrogen-bond geometry (Å, °)

*D*—H⋯*A*	*D*—H	H⋯*A*	*D*⋯*A*	*D*—H⋯*A*
N1—H2⋯N2^i^	0.89 (3)	2.14 (3)	3.014 (3)	168 (3)

Symmetry code: (i) 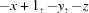.

**Fig. 1.**
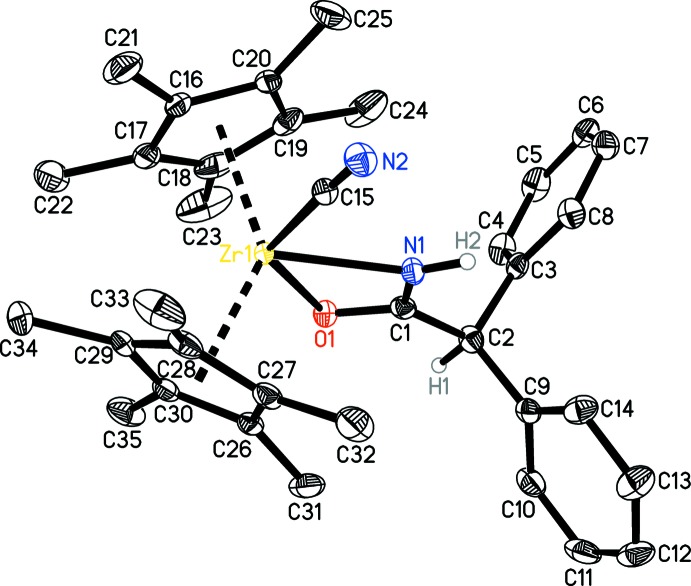
The molecular structure of the title compound with 30% displacement ellipsoids. Hydrogen atoms (except H1 and H2) are omitted for clarity.

## supplementary crystallographic information

### 1. Comment

We studied the reactions of several nitriles with metallocene precursors as Cp*_2_*M*(*η*^2^-Me_3_SiC_2_SiMe_3_) (Cp* = *η*^5^– pentamethylcyclopentadienyl, *M* = Ti, Zr) to synthesize and characterize new strained metallacycles with heteroatoms. In the reaction with diphenylacetonitrile the complex Cp*_2_Zr(N=CH-CHPh_2_)(N=C=CPh_2_) (Becker *et al.* (2013)) was formed after 4 h at 358 K. However a longer reaction time of 24 h led to the cleavage of the Ph_2_CH—CN bond upon partial oxidation thus forming the title compound. The zirconium(IV) atom is coordinated by two pentamethylcyclopentadienyl ligands in a *η*^5^-fashion, as well as by the N– and O-atoms of the amidate and an additional C≡N ligand. The four-membered ring is almost planar (mean deviation from the best plane defined by Zr1, O1, C1, N1 = 0.008 Å). The bond lengths O1—C1 1.290 (3) and N1—C1 1.294 (3) Å suggest the resonance form with electronic delocalization within the OCN unit. The molecules are connected to centrosymmetric dimers *via* N—H···N hydrogen bonds.

### 2. Experimental

To a stirred solution of Cp*_2_Zr(*η*^2^-Me_3_SiC_2_SiMe_3_) (0.266 g, 0.5 mmol) in 5 mL of toluene was added a solution of Ph_2_CH—CN (0.193 g, 1.0 mmol) in 10 mL of toluene. The solution was stirred for 2 h at room temperature and subsequently 24 h at 358 K till the color turned to orange. All volatiles were removed under vacuum and the residue was dissolved in *n*-hexane. After filtration the solution was allowed to stand at r. t.. Yellow crystals of the title compound were formed within 14 days. MS: *m/z* (CI): 596 [*M*]^+^ (3), 570 [M—CN]^+^ (1), 386 [Cp*_2_Zr(CN)]^+^ (1), 360 [Cp*_2_Zr]^+^ (1). IR: (ATR, cm^-1^): *υ* = 2900(w), 2133(vw), 1426 (*m*), 1259(*s*), 1019 (*vs*), 795(*vs*).

### 3. Refinement

H1 and H2 could be found from the difference Fourier map and were refined freely. All other H atoms were placed in idealized positions with d(C—H) = 0.98 (CH_3_) and 0.95 Å (CH) and refined using a riding model with *U*_iso_(H) fixed at 1.5 *U*_eq_(C) for CH_3_ and 1.2 *U*_eq_(C) for CH. In one of the both phenyl rings two distances (C5—C6 and C6—C7) were restrained to be equal (SADI).

### Crystal data

**Table:**

[Zr(C_10_H_15_)_2_(C_14_H_12_NO)(CN)]	*F*(000) = 1256
*M**_r_* = 597.93	*D*_x_ = 1.312 Mg m^−^^3^
Monoclinic, *P*2_1_/*n*	Mo *K*α radiation, λ = 0.71073 Å
Hall symbol: -P 2yn	Cell parameters from 9874 reflections
*a* = 11.8961 (3) Å	θ = 2.4–27.8°
*b* = 12.0640 (3) Å	µ = 0.39 mm^−^^1^
*c* = 21.2489 (5) Å	*T* = 150 K
β = 97.036 (1)°	Prism, red
*V* = 3026.56 (13) Å^3^	0.26 × 0.13 × 0.10 mm
*Z* = 4	

### Data collection

**Table:**

Bruker Kappa APEXII DUO diffractometer	6612 independent reflections
Radiation source: fine-focus sealed tube	5575 reflections with *I* > 2σ(*I*)
Curved graphite monochromator	*R*_int_ = 0.047
Detector resolution: 8.3333 pixels mm^-1^	θ_max_ = 27.0°, θ_min_ = 1.9°
φ and ω scans	*h* = −15→15
Absorption correction: multi-scan (*SADABS*; Bruker, 2008)	*k* = −15→15
*T*_min_ = 0.92, *T*_max_ = 1.00	*l* = −27→26
82697 measured reflections	

### Refinement

**Table:**

Refinement on *F*^2^	Primary atom site location: structure-invariant direct methods
Least-squares matrix: full	Secondary atom site location: difference Fourier map
*R*[*F*^2^ > 2σ(*F*^2^)] = 0.033	Hydrogen site location: inferred from neighbouring sites
*wR*(*F*^2^) = 0.085	H atoms treated by a mixture of independent and constrained refinement
*S* = 1.03	*w* = 1/[σ^2^(*F*_o_^2^) + (0.0376*P*)^2^ + 3.2391*P*] where *P* = (*F*_o_^2^ + 2*F*_c_^2^)/3
6612 reflections	(Δ/σ)_max_ = 0.001
370 parameters	Δρ_max_ = 1.11 e Å^−^^3^
1 restraint	Δρ_min_ = −0.46 e Å^−^^3^

### Special details

**Table:**

Geometry. All e.s.d.'s (except the e.s.d. in the dihedral angle between two l.s. planes) are estimated using the full covariance matrix. The cell e.s.d.'s are taken into account individually in the estimation of e.s.d.'s in distances, angles and torsion angles; correlations between e.s.d.'s in cell parameters are only used when they are defined by crystal symmetry. An approximate (isotropic) treatment of cell e.s.d.'s is used for estimating e.s.d.'s involving l.s. planes.
Refinement. Refinement of *F*^2^ against ALL reflections. The weighted *R*-factor *wR* and goodness of fit *S* are based on *F*^2^, conventional *R*-factors *R* are based on *F*, with *F* set to zero for negative *F*^2^. The threshold expression of *F*^2^ > σ(*F*^2^) is used only for calculating *R*-factors(gt) *etc*. and is not relevant to the choice of reflections for refinement. *R*-factors based on *F*^2^ are statistically about twice as large as those based on *F*, and *R*- factors based on ALL data will be even larger.

### Fractional atomic coordinates and isotropic or equivalent isotropic displacement parameters (Å^2^)

**Table:**

	*x*	*y*	*z*	*U*_iso_*/*U*_eq_	
N2	0.34293 (17)	−0.01561 (17)	−0.00689 (9)	0.0328 (4)	
C1	0.57641 (17)	0.23133 (18)	0.13538 (10)	0.0249 (4)	
C2	0.69873 (18)	0.27295 (19)	0.15050 (11)	0.0287 (5)	
C3	0.77338 (17)	0.18091 (19)	0.18260 (10)	0.0263 (4)	
C4	0.8135 (2)	0.1921 (2)	0.24651 (12)	0.0380 (6)	
H4	0.7986	0.2582	0.2683	0.046*	
C5	0.8753 (2)	0.1074 (2)	0.27887 (12)	0.0440 (7)	
H5	0.9026	0.1160	0.3225	0.053*	
C6	0.89672 (19)	0.0113 (2)	0.24769 (10)	0.0385 (6)	
H6	0.9376	−0.0473	0.2699	0.046*	
C7	0.85877 (19)	0.0001 (2)	0.18414 (11)	0.0361 (5)	
H7	0.8743	−0.0660	0.1625	0.043*	
C8	0.79802 (19)	0.0848 (2)	0.15157 (12)	0.0327 (5)	
H8	0.7731	0.0767	0.1076	0.039*	
C9	0.74590 (17)	0.32921 (19)	0.09490 (11)	0.0288 (5)	
C10	0.7888 (2)	0.4362 (2)	0.10502 (14)	0.0395 (6)	
H10	0.7859	0.4708	0.1450	0.047*	
C11	0.8354 (2)	0.4929 (2)	0.05789 (17)	0.0500 (8)	
H11	0.8649	0.5654	0.0658	0.060*	
C12	0.8391 (2)	0.4447 (2)	−0.00014 (15)	0.0476 (7)	
H12	0.8710	0.4835	−0.0326	0.057*	
C13	0.7963 (2)	0.3402 (3)	−0.01104 (13)	0.0462 (7)	
H13	0.7982	0.3069	−0.0514	0.055*	
C14	0.7503 (2)	0.2821 (2)	0.03594 (12)	0.0376 (6)	
H14	0.7216	0.2094	0.0276	0.045*	
C15	0.34628 (17)	0.04561 (18)	0.03489 (10)	0.0251 (4)	
C16	0.24386 (18)	0.02069 (19)	0.16252 (11)	0.0302 (5)	
C17	0.2474 (2)	0.1093 (2)	0.20580 (12)	0.0366 (6)	
C18	0.3598 (3)	0.1224 (2)	0.23301 (11)	0.0432 (7)	
C19	0.4268 (2)	0.0400 (2)	0.20780 (12)	0.0367 (6)	
C20	0.35450 (19)	−0.02379 (18)	0.16495 (11)	0.0294 (5)	
C21	0.1403 (2)	−0.0292 (3)	0.12447 (16)	0.0520 (8)	
H21A	0.1596	−0.0533	0.0831	0.078*	
H21B	0.0800	0.0265	0.1184	0.078*	
H21C	0.1143	−0.0929	0.1473	0.078*	
C22	0.1453 (3)	0.1632 (3)	0.22946 (17)	0.0641 (10)	
H22A	0.1349	0.1329	0.2711	0.096*	
H22B	0.0776	0.1482	0.1995	0.096*	
H22C	0.1573	0.2435	0.2330	0.096*	
C23	0.3975 (4)	0.2020 (3)	0.28566 (14)	0.0799 (13)	
H23A	0.3571	0.2724	0.2779	0.120*	
H23B	0.4792	0.2148	0.2874	0.120*	
H23C	0.3809	0.1708	0.3261	0.120*	
C24	0.5490 (2)	0.0163 (3)	0.22950 (18)	0.0754 (13)	
H24A	0.5546	−0.0385	0.2638	0.113*	
H24B	0.5870	0.0849	0.2449	0.113*	
H24C	0.5852	−0.0130	0.1940	0.113*	
C25	0.3906 (3)	−0.1274 (2)	0.13436 (15)	0.0571 (9)	
H25A	0.4570	−0.1117	0.1127	0.086*	
H25B	0.3286	−0.1542	0.1035	0.086*	
H25C	0.4100	−0.1842	0.1669	0.086*	
C26	0.34767 (17)	0.36419 (18)	0.06928 (10)	0.0254 (4)	
C27	0.31732 (19)	0.29185 (19)	0.01829 (10)	0.0286 (5)	
C28	0.2112 (2)	0.24409 (19)	0.02668 (12)	0.0321 (5)	
C29	0.17714 (18)	0.28634 (19)	0.08314 (12)	0.0304 (5)	
C30	0.26353 (19)	0.35821 (18)	0.11072 (11)	0.0272 (5)	
C31	0.4448 (2)	0.4441 (2)	0.07480 (14)	0.0426 (6)	
H31A	0.5096	0.4099	0.0579	0.064*	
H31B	0.4662	0.4633	0.1195	0.064*	
H31C	0.4223	0.5114	0.0507	0.064*	
C32	0.3803 (3)	0.2783 (3)	−0.03833 (13)	0.0503 (8)	
H32A	0.3611	0.3394	−0.0680	0.075*	
H32B	0.3590	0.2077	−0.0594	0.075*	
H32C	0.4620	0.2787	−0.0245	0.075*	
C33	0.1360 (3)	0.1774 (2)	−0.02120 (16)	0.0569 (9)	
H33A	0.0978	0.1196	0.0006	0.085*	
H33B	0.1819	0.1431	−0.0512	0.085*	
H33C	0.0793	0.2262	−0.0443	0.085*	
C34	0.0582 (2)	0.2786 (3)	0.10029 (18)	0.0566 (9)	
H34A	0.0069	0.3209	0.0696	0.085*	
H34B	0.0559	0.3090	0.1429	0.085*	
H34C	0.0344	0.2008	0.0995	0.085*	
C35	0.2603 (3)	0.4316 (2)	0.16760 (13)	0.0468 (7)	
H35A	0.3304	0.4224	0.1965	0.070*	
H35B	0.1954	0.4113	0.1895	0.070*	
H35C	0.2529	0.5091	0.1539	0.070*	
O1	0.50256 (12)	0.27068 (13)	0.16907 (7)	0.0267 (3)	
N1	0.53651 (15)	0.15917 (15)	0.09345 (9)	0.0255 (4)	
Zr1	0.357855 (15)	0.171301 (16)	0.116794 (9)	0.01859 (7)	
H1	0.696 (2)	0.334 (2)	0.1837 (13)	0.036 (7)*	
H2	0.580 (2)	0.125 (2)	0.0680 (13)	0.040 (8)*	

### Atomic displacement parameters (Å^2^)

**Table:**

	*U*^11^	*U*^22^	*U*^33^	*U*^12^	*U*^13^	*U*^23^
N2	0.0342 (10)	0.0311 (10)	0.0329 (11)	0.0002 (8)	0.0028 (8)	−0.0078 (9)
C1	0.0241 (10)	0.0236 (10)	0.0260 (11)	0.0015 (8)	−0.0010 (8)	0.0046 (9)
C2	0.0225 (10)	0.0283 (12)	0.0343 (12)	−0.0006 (9)	−0.0006 (9)	−0.0053 (10)
C3	0.0170 (9)	0.0332 (12)	0.0282 (11)	−0.0026 (8)	0.0017 (8)	0.0012 (9)
C4	0.0338 (12)	0.0481 (15)	0.0319 (13)	−0.0023 (11)	0.0032 (10)	−0.0060 (11)
C5	0.0350 (13)	0.0678 (19)	0.0278 (12)	0.0033 (13)	−0.0014 (10)	0.0094 (13)
C6	0.0257 (11)	0.0513 (16)	0.0385 (13)	0.0029 (11)	0.0031 (10)	0.0200 (12)
C7	0.0258 (11)	0.0368 (13)	0.0447 (14)	0.0020 (10)	0.0009 (10)	0.0025 (11)
C8	0.0266 (11)	0.0385 (13)	0.0315 (12)	0.0008 (10)	−0.0024 (9)	−0.0025 (10)
C9	0.0155 (9)	0.0278 (11)	0.0415 (13)	0.0017 (8)	−0.0028 (8)	0.0055 (10)
C10	0.0275 (11)	0.0281 (12)	0.0616 (17)	0.0028 (10)	−0.0004 (11)	−0.0026 (12)
C11	0.0345 (13)	0.0229 (12)	0.090 (2)	−0.0003 (10)	−0.0033 (14)	0.0133 (14)
C12	0.0319 (13)	0.0497 (16)	0.0590 (18)	−0.0039 (12)	−0.0038 (12)	0.0322 (15)
C13	0.0412 (14)	0.0571 (18)	0.0385 (14)	−0.0104 (13)	−0.0020 (11)	0.0103 (13)
C14	0.0357 (13)	0.0349 (13)	0.0410 (14)	−0.0109 (10)	−0.0005 (11)	0.0026 (11)
C15	0.0218 (10)	0.0241 (10)	0.0296 (11)	0.0012 (8)	0.0038 (8)	0.0022 (9)
C16	0.0260 (10)	0.0280 (11)	0.0378 (13)	−0.0022 (9)	0.0086 (9)	0.0112 (10)
C17	0.0463 (14)	0.0310 (13)	0.0374 (13)	0.0044 (11)	0.0251 (11)	0.0093 (11)
C18	0.0728 (19)	0.0369 (14)	0.0213 (11)	−0.0169 (13)	0.0112 (12)	0.0030 (10)
C19	0.0283 (11)	0.0468 (15)	0.0338 (13)	−0.0054 (10)	−0.0016 (10)	0.0237 (11)
C20	0.0343 (11)	0.0244 (11)	0.0316 (12)	0.0044 (9)	0.0129 (9)	0.0110 (9)
C21	0.0371 (14)	0.0476 (17)	0.069 (2)	−0.0163 (12)	−0.0024 (13)	0.0180 (15)
C22	0.084 (2)	0.0479 (18)	0.072 (2)	0.0241 (17)	0.0557 (19)	0.0213 (16)
C23	0.138 (4)	0.073 (2)	0.0282 (15)	−0.050 (2)	0.0097 (19)	−0.0038 (15)
C24	0.0341 (15)	0.099 (3)	0.087 (3)	−0.0100 (16)	−0.0154 (15)	0.069 (2)
C25	0.093 (2)	0.0313 (14)	0.0528 (18)	0.0231 (15)	0.0334 (17)	0.0160 (13)
C26	0.0232 (10)	0.0228 (10)	0.0293 (11)	0.0040 (8)	−0.0003 (8)	0.0076 (9)
C27	0.0348 (12)	0.0279 (11)	0.0230 (11)	0.0109 (9)	0.0024 (9)	0.0070 (9)
C28	0.0324 (12)	0.0236 (11)	0.0364 (13)	0.0061 (9)	−0.0121 (10)	0.0009 (10)
C29	0.0216 (10)	0.0247 (11)	0.0450 (14)	0.0083 (9)	0.0040 (9)	0.0088 (10)
C30	0.0312 (11)	0.0208 (10)	0.0295 (11)	0.0083 (8)	0.0036 (9)	0.0042 (9)
C31	0.0368 (13)	0.0326 (13)	0.0561 (17)	−0.0063 (11)	−0.0032 (12)	0.0183 (12)
C32	0.073 (2)	0.0499 (17)	0.0317 (14)	0.0236 (15)	0.0221 (13)	0.0135 (12)
C33	0.0587 (18)	0.0383 (15)	0.0634 (19)	0.0060 (13)	−0.0336 (16)	−0.0080 (14)
C34	0.0244 (12)	0.0531 (18)	0.094 (2)	0.0121 (12)	0.0138 (14)	0.0289 (17)
C35	0.073 (2)	0.0287 (13)	0.0398 (15)	0.0130 (13)	0.0124 (14)	−0.0039 (11)
O1	0.0238 (7)	0.0297 (8)	0.0266 (8)	0.0004 (6)	0.0030 (6)	−0.0016 (6)
N1	0.0214 (8)	0.0266 (9)	0.0290 (9)	0.0017 (7)	0.0047 (7)	−0.0027 (8)
Zr1	0.01732 (10)	0.01876 (10)	0.02010 (10)	0.00190 (7)	0.00397 (7)	0.00094 (8)

### Geometric parameters (Å, º)

**Table:**

N2—C15	1.152 (3)	C21—H21B	0.9800
C1—O1	1.290 (3)	C21—H21C	0.9800
C1—N1	1.294 (3)	C22—H22A	0.9800
C1—C2	1.536 (3)	C22—H22B	0.9800
C1—Zr1	2.681 (2)	C22—H22C	0.9800
C2—C9	1.528 (3)	C23—H23A	0.9800
C2—C3	1.529 (3)	C23—H23B	0.9800
C2—H1	1.03 (3)	C23—H23C	0.9800
C3—C8	1.383 (3)	C24—H24A	0.9800
C3—C4	1.390 (3)	C24—H24B	0.9800
C4—C5	1.389 (4)	C24—H24C	0.9800
C4—H4	0.9500	C25—H25A	0.9800
C5—C6	1.375 (3)	C25—H25B	0.9800
C5—H5	0.9500	C25—H25C	0.9800
C6—C7	1.377 (2)	C26—C27	1.404 (3)
C6—H6	0.9500	C26—C30	1.414 (3)
C7—C8	1.386 (3)	C26—C31	1.499 (3)
C7—H7	0.9500	C26—Zr1	2.533 (2)
C8—H8	0.9500	C27—C28	1.419 (3)
C9—C14	1.383 (4)	C27—C32	1.502 (3)
C9—C10	1.395 (3)	C27—Zr1	2.546 (2)
C10—C11	1.383 (4)	C28—C29	1.408 (4)
C10—H10	0.9500	C28—C33	1.503 (3)
C11—C12	1.369 (4)	C28—Zr1	2.581 (2)
C11—H11	0.9500	C29—C30	1.415 (3)
C12—C13	1.369 (4)	C29—C34	1.507 (3)
C12—H12	0.9500	C29—Zr1	2.585 (2)
C13—C14	1.386 (4)	C30—C35	1.503 (3)
C13—H13	0.9500	C30—Zr1	2.515 (2)
C14—H14	0.9500	C31—H31A	0.9800
C15—Zr1	2.300 (2)	C31—H31B	0.9800
C16—C17	1.407 (4)	C31—H31C	0.9800
C16—C20	1.416 (3)	C32—H32A	0.9800
C16—C21	1.513 (4)	C32—H32B	0.9800
C16—Zr1	2.531 (2)	C32—H32C	0.9800
C17—C18	1.399 (4)	C33—H33A	0.9800
C17—C22	1.518 (4)	C33—H33B	0.9800
C17—Zr1	2.544 (2)	C33—H33C	0.9800
C18—C19	1.419 (4)	C34—H34A	0.9800
C18—C23	1.501 (4)	C34—H34B	0.9800
C18—Zr1	2.536 (2)	C34—H34C	0.9800
C19—C20	1.403 (4)	C35—H35A	0.9800
C19—C24	1.497 (4)	C35—H35B	0.9800
C19—Zr1	2.556 (2)	C35—H35C	0.9800
C20—C25	1.496 (4)	O1—Zr1	2.2729 (15)
C20—Zr1	2.569 (2)	N1—Zr1	2.2455 (18)
C21—H21A	0.9800	N1—H2	0.89 (3)
			
O1—C1—N1	114.36 (19)	C32—C27—Zr1	122.23 (16)
O1—C1—C2	117.13 (19)	C29—C28—C27	108.3 (2)
N1—C1—C2	128.5 (2)	C29—C28—C33	124.1 (2)
O1—C1—Zr1	57.79 (10)	C27—C28—C33	126.7 (3)
N1—C1—Zr1	56.59 (11)	C29—C28—Zr1	74.33 (13)
C2—C1—Zr1	174.91 (16)	C27—C28—Zr1	72.56 (12)
C9—C2—C3	114.79 (18)	C33—C28—Zr1	127.75 (16)
C9—C2—C1	114.33 (18)	C28—C29—C30	107.7 (2)
C3—C2—C1	109.85 (18)	C28—C29—C34	124.1 (2)
C9—C2—H1	104.9 (15)	C30—C29—C34	126.4 (2)
C3—C2—H1	106.4 (15)	C28—C29—Zr1	74.05 (12)
C1—C2—H1	105.7 (15)	C30—C29—Zr1	71.18 (12)
C8—C3—C4	118.6 (2)	C34—C29—Zr1	132.34 (17)
C8—C3—C2	122.8 (2)	C26—C30—C29	107.9 (2)
C4—C3—C2	118.5 (2)	C26—C30—C35	124.1 (2)
C5—C4—C3	120.7 (2)	C29—C30—C35	127.2 (2)
C5—C4—H4	119.7	C26—C30—Zr1	74.46 (12)
C3—C4—H4	119.7	C29—C30—Zr1	76.63 (12)
C6—C5—C4	120.0 (2)	C35—C30—Zr1	122.86 (16)
C6—C5—H5	120.0	C26—C31—H31A	109.5
C4—C5—H5	120.0	C26—C31—H31B	109.5
C5—C6—C7	119.8 (2)	H31A—C31—H31B	109.5
C5—C6—H6	120.1	C26—C31—H31C	109.5
C7—C6—H6	120.1	H31A—C31—H31C	109.5
C6—C7—C8	120.4 (2)	H31B—C31—H31C	109.5
C6—C7—H7	119.8	C27—C32—H32A	109.5
C8—C7—H7	119.8	C27—C32—H32B	109.5
C3—C8—C7	120.5 (2)	H32A—C32—H32B	109.5
C3—C8—H8	119.7	C27—C32—H32C	109.5
C7—C8—H8	119.7	H32A—C32—H32C	109.5
C14—C9—C10	117.8 (2)	H32B—C32—H32C	109.5
C14—C9—C2	125.2 (2)	C28—C33—H33A	109.5
C10—C9—C2	117.0 (2)	C28—C33—H33B	109.5
C11—C10—C9	121.1 (3)	H33A—C33—H33B	109.5
C11—C10—H10	119.4	C28—C33—H33C	109.5
C9—C10—H10	119.4	H33A—C33—H33C	109.5
C12—C11—C10	120.2 (2)	H33B—C33—H33C	109.5
C12—C11—H11	119.9	C29—C34—H34A	109.5
C10—C11—H11	119.9	C29—C34—H34B	109.5
C11—C12—C13	119.4 (3)	H34A—C34—H34B	109.5
C11—C12—H12	120.3	C29—C34—H34C	109.5
C13—C12—H12	120.3	H34A—C34—H34C	109.5
C12—C13—C14	121.0 (3)	H34B—C34—H34C	109.5
C12—C13—H13	119.5	C30—C35—H35A	109.5
C14—C13—H13	119.5	C30—C35—H35B	109.5
C9—C14—C13	120.5 (2)	H35A—C35—H35B	109.5
C9—C14—H14	119.8	C30—C35—H35C	109.5
C13—C14—H14	119.8	H35A—C35—H35C	109.5
N2—C15—Zr1	177.97 (19)	H35B—C35—H35C	109.5
C17—C16—C20	108.1 (2)	C1—O1—Zr1	93.51 (12)
C17—C16—C21	127.5 (2)	C1—N1—Zr1	94.67 (14)
C20—C16—C21	124.1 (2)	C1—N1—H2	122.6 (18)
C17—C16—Zr1	74.40 (13)	Zr1—N1—H2	142.7 (18)
C20—C16—Zr1	75.33 (12)	N1—Zr1—O1	57.43 (6)
C21—C16—Zr1	121.31 (17)	N1—Zr1—C15	76.14 (7)
C18—C17—C16	107.8 (2)	O1—Zr1—C15	133.57 (6)
C18—C17—C22	125.3 (3)	N1—Zr1—C30	118.39 (7)
C16—C17—C22	125.5 (3)	O1—Zr1—C30	81.87 (6)
C18—C17—Zr1	73.72 (13)	C15—Zr1—C30	124.57 (7)
C16—C17—Zr1	73.40 (12)	N1—Zr1—C16	127.16 (7)
C22—C17—Zr1	128.88 (17)	O1—Zr1—C16	126.89 (7)
C17—C18—C19	108.5 (2)	C15—Zr1—C16	80.32 (8)
C17—C18—C23	124.3 (3)	C30—Zr1—C16	113.99 (7)
C19—C18—C23	126.8 (3)	N1—Zr1—C26	88.41 (7)
C17—C18—Zr1	74.31 (14)	O1—Zr1—C26	72.94 (6)
C19—C18—Zr1	74.59 (13)	C15—Zr1—C26	107.97 (7)
C23—C18—Zr1	122.88 (19)	C30—Zr1—C26	32.52 (7)
C20—C19—C18	107.5 (2)	C16—Zr1—C26	144.15 (7)
C20—C19—C24	126.2 (3)	N1—Zr1—C18	107.68 (9)
C18—C19—C24	125.9 (3)	O1—Zr1—C18	74.38 (7)
C20—C19—Zr1	74.59 (13)	C15—Zr1—C18	125.16 (8)
C18—C19—Zr1	73.05 (14)	C30—Zr1—C18	102.10 (9)
C24—C19—Zr1	124.10 (17)	C16—Zr1—C18	53.18 (8)
C19—C20—C16	108.0 (2)	C26—Zr1—C18	126.59 (8)
C19—C20—C25	123.6 (2)	N1—Zr1—C17	137.22 (8)
C16—C20—C25	128.0 (3)	O1—Zr1—C17	102.76 (7)
C19—C20—Zr1	73.63 (13)	C15—Zr1—C17	112.35 (8)
C16—C20—Zr1	72.43 (12)	C30—Zr1—C17	91.95 (8)
C25—C20—Zr1	124.99 (16)	C16—Zr1—C17	32.20 (8)
C16—C21—H21A	109.5	C26—Zr1—C17	124.23 (7)
C16—C21—H21B	109.5	C18—Zr1—C17	31.98 (9)
H21A—C21—H21B	109.5	N1—Zr1—C27	86.83 (7)
C16—C21—H21C	109.5	O1—Zr1—C27	98.99 (7)
H21A—C21—H21C	109.5	C15—Zr1—C27	76.51 (8)
H21B—C21—H21C	109.5	C30—Zr1—C27	53.65 (7)
C17—C22—H22A	109.5	C16—Zr1—C27	132.01 (8)
C17—C22—H22B	109.5	C26—Zr1—C27	32.08 (7)
H22A—C22—H22B	109.5	C18—Zr1—C27	155.74 (9)
C17—C22—H22C	109.5	C17—Zr1—C27	135.74 (8)
H22A—C22—H22C	109.5	N1—Zr1—C19	84.38 (7)
H22B—C22—H22C	109.5	O1—Zr1—C19	78.67 (7)
C18—C23—H23A	109.5	C15—Zr1—C19	98.49 (9)
C18—C23—H23B	109.5	C30—Zr1—C19	133.98 (8)
H23A—C23—H23B	109.5	C16—Zr1—C19	53.28 (7)
C18—C23—H23C	109.5	C26—Zr1—C19	150.00 (8)
H23A—C23—H23C	109.5	C18—Zr1—C19	32.36 (9)
H23B—C23—H23C	109.5	C17—Zr1—C19	53.30 (8)
C19—C24—H24A	109.5	C27—Zr1—C19	170.73 (8)
C19—C24—H24B	109.5	N1—Zr1—C20	95.14 (7)
H24A—C24—H24B	109.5	O1—Zr1—C20	109.66 (7)
C19—C24—H24C	109.5	C15—Zr1—C20	72.23 (7)
H24A—C24—H24C	109.5	C30—Zr1—C20	144.43 (7)
H24B—C24—H24C	109.5	C16—Zr1—C20	32.24 (7)
C20—C25—H25A	109.5	C26—Zr1—C20	176.37 (7)
C20—C25—H25B	109.5	C18—Zr1—C20	52.96 (8)
H25A—C25—H25B	109.5	C17—Zr1—C20	53.11 (7)
C20—C25—H25C	109.5	C27—Zr1—C20	147.17 (8)
H25A—C25—H25C	109.5	C19—Zr1—C20	31.78 (8)
H25B—C25—H25C	109.5	N1—Zr1—C28	115.49 (8)
C27—C26—C30	108.32 (19)	O1—Zr1—C28	125.85 (7)
C27—C26—C31	125.7 (2)	C15—Zr1—C28	71.97 (7)
C30—C26—C31	125.6 (2)	C30—Zr1—C28	53.14 (7)
C27—C26—Zr1	74.44 (13)	C16—Zr1—C28	100.58 (8)
C30—C26—Zr1	73.02 (12)	C26—Zr1—C28	52.91 (7)
C31—C26—Zr1	124.11 (15)	C18—Zr1—C28	136.57 (9)
C26—C27—C28	107.7 (2)	C17—Zr1—C28	106.73 (8)
C26—C27—C32	125.4 (2)	C27—Zr1—C28	32.12 (8)
C28—C27—C32	126.5 (2)	C19—Zr1—C28	153.78 (8)
C26—C27—Zr1	73.47 (12)	C20—Zr1—C28	124.41 (8)
C28—C27—Zr1	75.32 (13)		

### Hydrogen-bond geometry (Å, º)

**Table:**

*D*—H···*A*	*D*—H	H···*A*	*D*···*A*	*D*—H···*A*
N1—H2···N2^i^	0.89 (3)	2.14 (3)	3.014 (3)	168 (3)

Symmetry code: (i) −*x*+1, −*y*, −*z*.
